# Dan-Shen-Yin Granules Prevent Hypoxia-Induced Pulmonary Hypertension *via* STAT3/HIF-1α/VEGF and FAK/AKT Signaling Pathways

**DOI:** 10.3389/fphar.2022.844400

**Published:** 2022-03-11

**Authors:** Ran-Ran Wang, Tian-Yi Yuan, Di Chen, Yu-Cai Chen, Shu-Chan Sun, Shou-Bao Wang, Ling-Lei Kong, Lian-Hua Fang, Guan-Hua Du

**Affiliations:** ^1^ State Key Laboratory of Bioactive Substances and Function of Natural Medicines, Institute of Materia Medica, Chinese Academy of Medical Sciences and Peking Union Medical College, Beijing, China; ^2^ School of Traditional Chinese Medicine, Beijing University of Chinese Medicine, Beijing, China

**Keywords:** traditional chinese medicine, Dan-Shen-Yin, hypoxia-induced pulmonary hypertension, network pharmacology, mechanism

## Abstract

Traditional Chinese medicine (TCM) plays an important role in the treatment of complex diseases, especially cardiovascular diseases. However, it is hard to identify their modes of action on account of their multiple components. The present study aims to evaluate the effects of Dan-Shen-Yin (DSY) granules on hypoxia-induced pulmonary hypertension (HPH), and then to decipher the molecular mechanisms of DSY. Systematic pharmacology was employed to identify the targets of DSY on HPH. Furthermore, core genes were identified by constructing a protein-protein interaction (PPI) network and analyzed by Gene Ontology (GO) and Kyoto Encyclopedia of Genes (KEGG) analysis. Related genes and pathways were verified using a hypoxia-induced mouse model and hypoxia-treated pulmonary artery cells. Based on network pharmacology, 147 potential targets of DSY on HPH were found, constructing a PPI network, and 13 hub genes were predicted. The results showed that the effect of DSY may be closely associated with AKT serine/threonine kinase 1 (AKT1), signal transducer and activator of transcription 3 (STAT3), and HIF-1 signaling pathways, as well as biological processes such as cell proliferation. Consistent with network pharmacology analysis, experiments *in vivo* demonstrated that DSY could prevent the development of HPH in a hypoxia-induced mouse model and alleviate pulmonary vascular remodeling. In addition, inhibition of STAT3/HIF-1α/VEGF and FAK/AKT signaling pathways might serve as mechanisms. Taken together, the network pharmacology analysis suggested that DSY exhibited therapeutic effects through multiple targets in the treatment of HPH. The inferences were initially confirmed by subsequent *in vivo* and *in vitro* studies. This study provides a novel perspective for studying the relevance of TCM and disease processes and illustrates the advantage of this approach and the multitargeted anti-HPH effect of DSY.

## Introduction

Pulmonary hypertension (PH) is one of the most serious chronic severe cardiopulmonary dysfunctional diseases, characterized by progressive remodeling of pulmonary arteries, resulting in elevated pulmonary vascular resistance and, eventually, in right ventricular failure and death (McLaughlin, 2011; Lau et al., 2017; Simonneau et al., 2019). PH due to lung diseases and/or hypoxia is one of the five types of PH. Chronic exposure to hypoxia induces an inflammatory response in the lung that could contribute to hypoxic vasoconstriction and remodeling (Lee et al., 2012). The pathological mechanisms of hypoxia-induced pulmonary hypertension (HPH) frequently involve smooth muscle cell proliferation, muscularization of precapillary arterioles, loss of distal pulmonary vessels, endothelial-to-mesenchymal transition, mitochondrial-induced apoptosis, and many other pathological and physiological processes (Wilkins et al., 2015; Rowan et al., 2016; Thompson and Lawrie, 2017; Marshall et al., 2018; Thenappan et al., 2018; Zhao et al., 2019). Although we have a better understanding of the pathophysiology and treatment of HPH, there is still no effective targeted drug for this disease (Lee et al., 2012; Hoeper et al., 2016b; Zhao et al., 2019). At present, the most commonly used drugs in clinical practice are mainly vasodilators, targeting voltage gated, L type calcium channels, nitric oxide cyclic guanosine monophosphate (cGMP), endothelin, and prostacyclin (Thenappan et al., 2018). Combination therapy has attracted increasingly attention and shown better therapeutic effect (Tapson et al., 2013; McLaughlin et al., 2015; Sitbon et al., 2015; Hoeper et al., 2016a). Several pathways have been reported to be involved in HPH, such as CD146-HIF-1α pathway (Luo et al., 2019), JAK2/STAT3 signaling pathway (Zhang et al., 2020) and so on. Considering the complex pathways involved in the pathogenesis of HPH and better effects of combination therapy, treatments targeting multiple targets may be more efficacious.

With a long history of thousands of years, traditional Chinese medicine (TCM) plays an important role in the treatment of complex diseases worldwide. With their respective dosages according to the guidance of Chinese medicine theory and the rule of “Jun-Chen-Zuo-Shi,” also known as “King, Vassal, Assistant and Delivery servant” (Yi and Chang, 2004), the TCM preparation was produced. However, the rather complex multi-ingredient components in natural medicine formulations hinder our ability to easily identify the molecular mechanisms of their efficacy.

With the rapid development of bioinformatics, network pharmacology has emerged as an effective and promising approach for highly multidimensional diseases (Kibble et al., 2015) and TCM with complex ingredients. Network pharmacology is an approach that encompasses systems biology, network analysis, connectivity, redundancy, and pleiotropy (Hopkins, 2008). It is capable of describing complex interactions among biological systems of the human body, drugs, and diseases from a network perspective (Zhang et al., 2017). Previous studies have demonstrated the effectiveness and advantages of network-based methods for the discovery of bioactive compounds and the elucidation of the mechanisms of action of herbal formulas (Li and Zhang, 2013; Boezio et al., 2017; Han et al., 2021).

Dan-Shen-Yin (DSY) is a well-known traditional Chinese formula comprising *Salvia miltiorrhiza Bunge* [Lamiaceae; Salviae miltiorrhizae radix et rhizoma], *Santalum album* L. [ Santalaceae; Santalum L.] and *Amomum villosum* Lour. It is widely used in clinical practice for the treatment of cardiovascular diseases and has produced a favorable effect (Yan et al., 2012). *S. Miltiorrhiza Bunge* is commonly used to improve body function, such as promoting blood circulation and restoring/enhancing (MEIm et al., 2019). It is reported that active ingredients in *S. Miltiorrhiza Bunge*, such as Tanshinone IIA and Salvianolic acid A, could effectively attenuate HPH (Zheng et al., 2015; Chen et al., 2016; Zhang et al., 2018; Ren et al., 2019). Moreover, ShengMai DanShenYin combined with general therapy has been reported to have a significant effect on the treatment of severe pulmonary bloating (Liu and Wang, 2017). DSY was also reported to be used to promote blood circulation and remove stasis in the treatment of two cases of acute exacerbation of chronic obstructive pulmonary disease (Wang, 2017). Furthermore, DSY combined with conventional treatment had a significant effect on chronic pulmonary heart disease and heart failure (Cai et al., 2015). Our previous study has found that DSY granules have a strong protective effect on cardiovascular system as well (Yan et al., 2021). Based on these studies, we explored whether DSY can prevent hypoxic pulmonary hypertension and the underlying mechanisms.

To elucidate the effects and molecular mechanisms of DSY on HPH, we performed a network pharmacology analysis and verified by experiments. Firstly, potential targets of DSY on HPH were identified by data mining. Next, we constructed a protein-protein interaction (PPI) network of potential targets, and the core genes were extracted. Then, a significant biological functional annotation for the identified targets helped to illustrate the potential anti-HPH mechanisms of DSY. The target-function interaction network was constructed and analyzed to identify key pathways. To validate the results, we estimated the protective effects and molecule mechanisms of DSY on hemodynamics and vascular remodeling in an HPH mouse model and hypoxia-treated pulmonary artery cells. The study utilized network-based techniques to investigate the natural compounds in DSY and to illustrate its predicted modes of action, which were validated by experiments *in vivo* and *in vitro*.

## Materials and Methods

### Bioactive Compounds and Targets of Dan-Shen-Yin Collection

We collected targets of DSY from different databases. Firstly, the TCMSP (Traditional Chinese Medicine Systems Pharmacology Database and Analysis Platform) database (Ru et al., 2014) was used to screen the bioactive ingredients of DSY. Anti-HPH components were then further identified using virtual screening of the components based on ADME parameters with oral bioavailability (OB) threshold OB ≥30% and drug similarity (DL) threshold DL ≥0.18 as parameters. For each compound, the chemical structure was acquired from PubChem. Then, putative targets were exacted from SwissTargetPrediction (Daina et al., 2019), DrugBank (Wishart et al., 2018), and TCMSP. The obtained targets were then mapped to UniProt (UniProt Consortium, 2021) for normalization.

### Hypoxia-Induced Pulmonary Hypertension Related Targets

HPH-related targets were collected from DisGeNET (Piñero et al., 2020) and GeneCards databases (Stelzer et al., 2016) with “Hypoxic pulmonary hypertension” and “Group 3 pulmonary hypertension” as keywords. Reproducible targets were removed, and the acquired targets were then mapped to UniProt (UniProt, 2021) for normalization.

### Construction and Analysis of Protein-Protein Interaction Network

The intersection of obtained drug targets and HPH-related targets were considered as potential targets of DSY for the treatment of HPH. These targets were uploaded with STRING 11.0 (Szklarczyk et al., 2019) to analyze PPI with a screening threshold as 0.9. The PPI network was then visualized using Cytoscape 3.7.2 (Shannon et al., 2003). The topological parameters in the network are calculated using the network analyzer plugin in Cytoscape. The 13 nodes with the highest ranking in terms of degree and betweenness centrality were considered as the core genes of the network.

### Gene Ontology and the Kyoto Encyclopedia of Genes and Genomes Enrichment

We utilized Gene Ontology (GO) enrichment and Kyoto Encyclopedia of Genes and Genomes (KEGG) pathway analysis for functional annotation. To annotate the role of candidate genes and proteins associated with HPH, the potential therapeutic targets of DSY were analyzed by DAVID (Huang da et al., 2009) as input to search for related biological processes, cellular components, molecular functions, and pathways. GO/KEGG terms with *p* < 0.05 were considered significantly enriched.

### Preparation of Dan-Shen-Yin Granules

*S. miltiorrhiza Bunge* [Lamiaceae; Salviae miltiorrhizae radix et rhizoma] (19030401, Shandong Jishi Pharmaceutical Co., Ltd., China), *S. album* L. [ Santalaceae; Santalum L.] (601002910, Beijing Tongrentang Wangfujing Hospital of Traditional Chinese Medicine Co., Ltd., China) and *A. villosum* Lour. [Zingiberaceae; Amomum Roxb.] (171206002, Beijing Tongrentang Wangfujing Hospital of Traditional Chinese Medicine Co., Ltd., China) were added in a mass ratio of 10:1:1. All of them were identified to comply with the relevant provisions of Chinese Pharmacopoeia (Part I) (2015 edition). Firstly, 5 times the weight of water were added to the drug and soaked for 2 h. After decocting for 1 h and filtering, dregs and filtrate were collected separately. Then, 4 times the weight of water was added to dregs got before. After decocting for 1 h and filtering, dregs and filtrate were collected again. Next, we added 3 times of water, decocted for 1 h, and filtered to collect the filtrate. Later, we distilled and concentrated all the colature under reduced pressure at 70°C and −0.05 MPa. The obtained concentrated liquid and medical auxiliary material starch were passed through a multifunctional fluidized bed and finally mixed evenly by a three-dimensional motion mixer to obtain DSY granules. Based on the total weight of the medicinal materials and the quality of the prepared granules, it is calculated that 1 g of DSY granules is equivalent to 1.26 g of crude drug (Yan et al., 2021).

### Animals

Forty male C57/6J mice (18–20 g) were provided by Beijing HFK Bioscience Co., Ltd. (Beijing, China). All animal experimental procedures were approved by the Institute of Animal Care and Use Committee, Chinese Academy of Medical Sciences. The animals were allowed to acclimate for 3 days in the facility, maintained at 22°C ± 3°C and 45% ± 10% humidity, following a regular 12 h/12 h light/dark schedule. All animals had free access to water and food.

Mice were randomly divided into the following four groups: group I (control), kept in normal environment for 3 weeks; group II (model), kept in normal environment for 1 week, and then exposed to 10% oxygen in a hypoxic chamber for the next 2 weeks; group III (Sildenafil), kept in normal environment for 1 week, and then exposed to 10% oxygen for next 2 weeks, with Sildenafil (30 mg/kg/d) orally for 3 weeks; group IV (DSY), kept in normal environment for 1 week, and then exposed to 10% oxygen for next 2 weeks, with DSY (4.6 g/kg/d, equivalent to the clinical dose) orally for 3 weeks. At the end of treatment, mice were anesthetized by tribromoethanol, and then the right ventricular systolic pressure (RVSP), lung, heart, and blood samples were collected from individual mice.

### Walking Distance of Exhausted Mice Detection

The 6-minute walk test (6MWT) is a commonly used test to evaluate the activity tolerance of patients with pulmonary hypertension (Agarwala and Salzman, 2020). Mice were placed on the treadmill to observe the walking distance until they were exhausted to reflect their cardiopulmonary function. If the mouse stayed on the electrode plate for more than 10 s, it was considered exhausted.

### Right Ventricular Systolic Pressure Measurement

Mice were anesthetized with tribromoethanol. A middle incision was made at the neck to expose the right external jugular vein. A Mikro-Tip pressure catheter (Millar, Houston, TX, United States) was inserted into the jugular vein and then into the right ventricle (RV) for monitoring RVSP. A stable ventricular pressure wave indicates the exact position of the catheter in RV. RVSP was recorded with PowerLab (AD Instruments, Colorado Springs, CO, United States) and analyzed with LabChart V8 software (AD Instruments, Colorado Springs, CO, United States).

### Organ Index Measurement and Specimen Preparation

Once hemodynamic data were collected, the blood was collected from the abdominal aorta. Then the mice died, and the organs were harvested. We recorded the weights of the whole heart, ventricle, right ventricle, lung, and body. The ratios of right ventricle-to-interventricular septal plus left ventricular weight (RV/LV + S), which was an indicator of right cardiac hypertrophy (RVH), were calculated.

### Morphometric Analysis of Lung

The left lung was isolated, immediately flushed with saline, and then fixed in 4% paraformaldehyde for 24 h. After being dehydrated and cleared, lungs were embedded in paraffin wax. The paraffin-embedded tissues were stained with Masson’s trichrome. The sections were observed under a light microscope for morphometric changes, and then photomicrographs were obtained.

### Cell Culturing

Human pulmonary artery endothelial cells (HPAECs) (Catalog#3100) and Human pulmonary smooth muscle cells (HPASMCs) (Catalog#3110) were purchased from ScienCell (Carlsbad, CA, United States). HPAECs were cultured in Endothelial Cell Medium (ECM) (ScienCell, Carlsbad, CA, United States) supplemented with Endothelial Cell Growth Supplement (ECGS) and 5% fetal bovine serum (FBS). HPASMCs were cultured in Dulbecco’s Modified Eagle Medium (DMEM) (Gibco, Aukland, New Zealand) supplemented with 10% FBS (Gibco, Aukland, New Zealand). The cells were cultured at 37°C with 5% CO_2_ in humidified conditions. All experiments were carried out with cells at passages of 4–8.

### Hypoxia-Induced Proliferation Assay

The HPAECs at passage of 4–6 were seeded into normal 96 well plates at a density of 5,000 cells/well. The HPASMCs at passage of 4–6 were cultured in normal 96 well plates at a density of 4,000 cells/well. After 24 h, the cells were changed to fresh serum-free medium for 24 h. Then, HPAECs and HPASMCs were treated with or without DSY (1, 3 and 10 μg/ml) for 2 h in the normal incubator. The cells in control groups were remained in normal conditions (21% O_2_, 5% CO_2_). The others were moved into the Tri-Gas CO_2_ incubator (Thermo, United States) containing humidified hypoxia gas (1% O_2_, 5% CO_2_). After 48 h incubation, the cell viability was detected by the CCK8 kit. Absorbance values were read at the wave of 450 nm by a Spectra Max M5 microplate reader (Molecular Device, San Jose, CA, United States).

### Western Blotting

HPAECs at passage of 5–8 were cultured in 60 mm dishes at a density of 1 × 10^5^/ml. HPASMC’s at passage of 5–8 were cultured in 60 mm dishes at a density of 5 × 10^4^/ml. When the cells reached the confluence of 80%, they were starved for 24 h.The cells treatment were the same as above and then incubated under a normal or hypoxic environment for 48 h.

The lung tissues and cells were homogenized on ice in RIPA lysis buffer supplemented with protease inhibitor cocktail. The mixture was centrifuged at 12,000 rpm for 20 min at 4°C, and then the supernatant was collected. Protein concentration was determined spectrophotometrically using a bicinchoninic acid protein assay with serial dilution of bovine serum albumin (BSA) as the standard. Protein samples were mixed with 5 × loading buffer and heated at 100°C for 10 min. Equal amounts of protein extracts were subjected to SDS-PAGE and electrophoretically transferred to a polyvinylidene difluoride (PVDF) membrane (IPVH00010, Millipore, Burlington, MA, United States). After being blocked with 5% BSA for 2 h at room temperature, membranes were incubated overnight at 4°C with the following primary antibodies: β-actin (1:2,000, Proteintech Group, United States), p-STAT3 (1:2,000, Cell Signaling Technology, Danvers, MA, United States), STAT3 (1:1,000, Cell Signaling Technology, Danvers, MA, United States), HIF-1α (1:200, Santa Cruz Biotechnology, Dallas, TX, United States), c-Myc (1:1,000, Cell Signaling Technology, United States), VEGF (1:1,000, Abcam, United States), p-FAK (1:1,000, Cell Signaling Technology, Danvers, MA, United States), p-Akt (1:1,000, Cell Signaling Technology, Danvers, MA, United States), Akt (1:1,000, Cell Signaling Technology, Danvers, MA, United States) and PCNA (1:1,000, Abcam, Waltham, MA, United States). The membranes were then washed three times for 15 min with TBS-0.5% Tween 20 and subsequently incubated with anti-rabbit IgG (1:5,000, Gene-Protein Link) or anti-mouse IgG (1:5,000, Gene-Protein Link) secondary antibodies for 2 h at room temperature. After washing four times with TBS-0.5% Tween 20 for 20 min, immunoreactivity bands were visualized by enhanced chemiluminescence (Tanon 5,200, Yuanpinghao Bio-tech, Beijing, China) and quantified using Image J software. Relative protein expression was normalized relative to β-actin.

### Statistical Analysis

Results are expressed as the mean ± standard error of the mean (SEM). The significance of the differences between groups was determined by one-way analysis of variance (ANOVA) followed by Tukey’s post hoc test as appropriate. Differences were considered statistically significant at *p*-values less than 0.05. The images in this article were created using GraphPad Prism 8 (GraphPad Software Inc., La Jolla, CA, United States).

## Results

**FIGURE 1:**
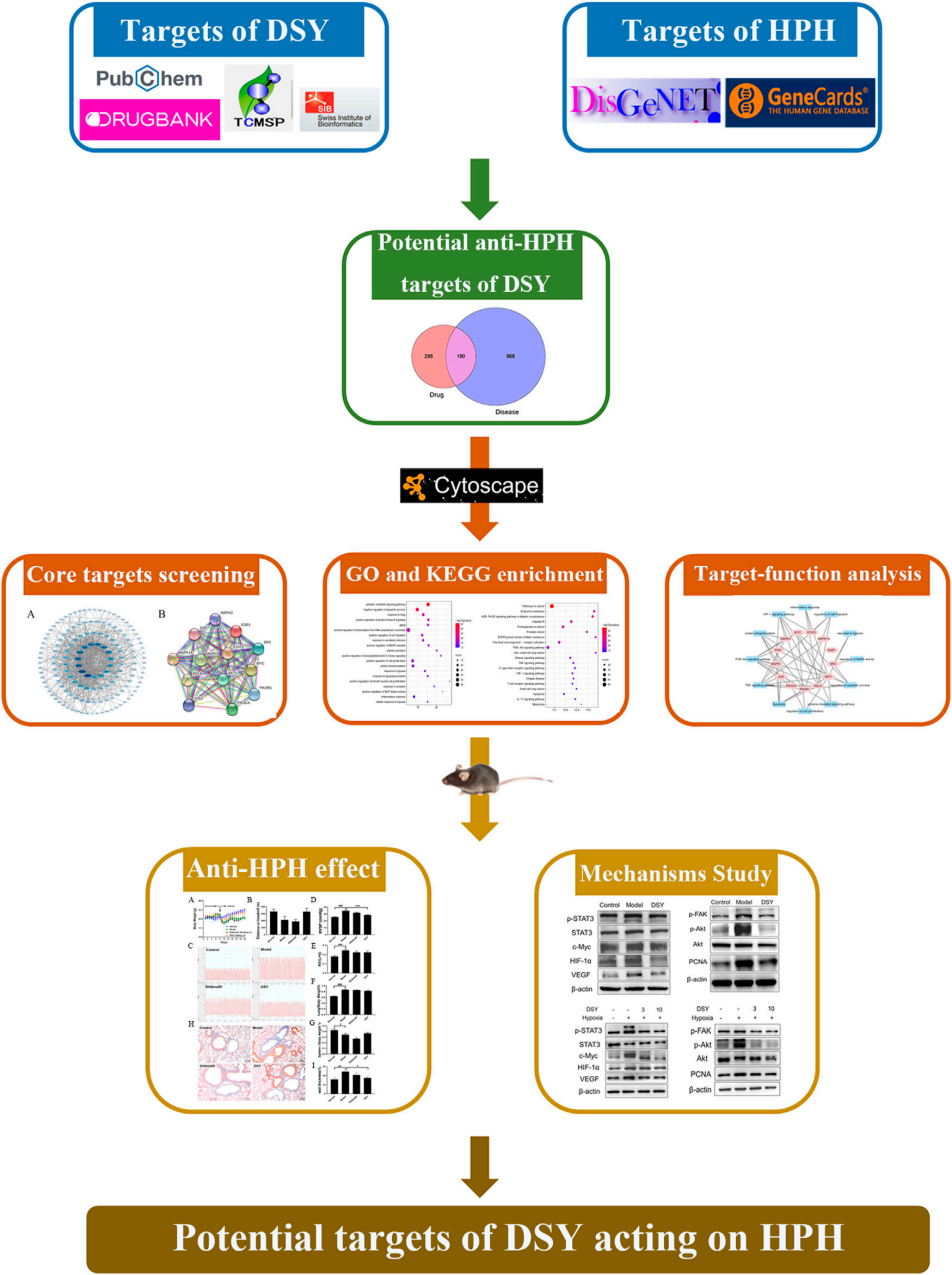
Workflow of the present study.

The whole workflow is illustrated in Figure 1.

### Identification of Dan-Shen-Yin’s Potential Therapeutic Targets by Intersection Analysis

78 active components of DSY were obtained through screening from the TCMSP database, including 60 components in *S. miltiorrhiza Bunge*, 3 components in *S. album* L., and 15 components in *A. villosum* Lour. Based on data mining, we identified 485 pharmacological targets of DSY, 190 of which also appeared in the data set of HPH-related targets. Therefore, these 190 targets were considered to be potential targets of DSY in the treatment of HPH and were further analyzed.

### Construction of Protein-Protein Interaction Network of Potential Therapeutic Targets of Dan-Shen-Yin

**FIGURE 2:**
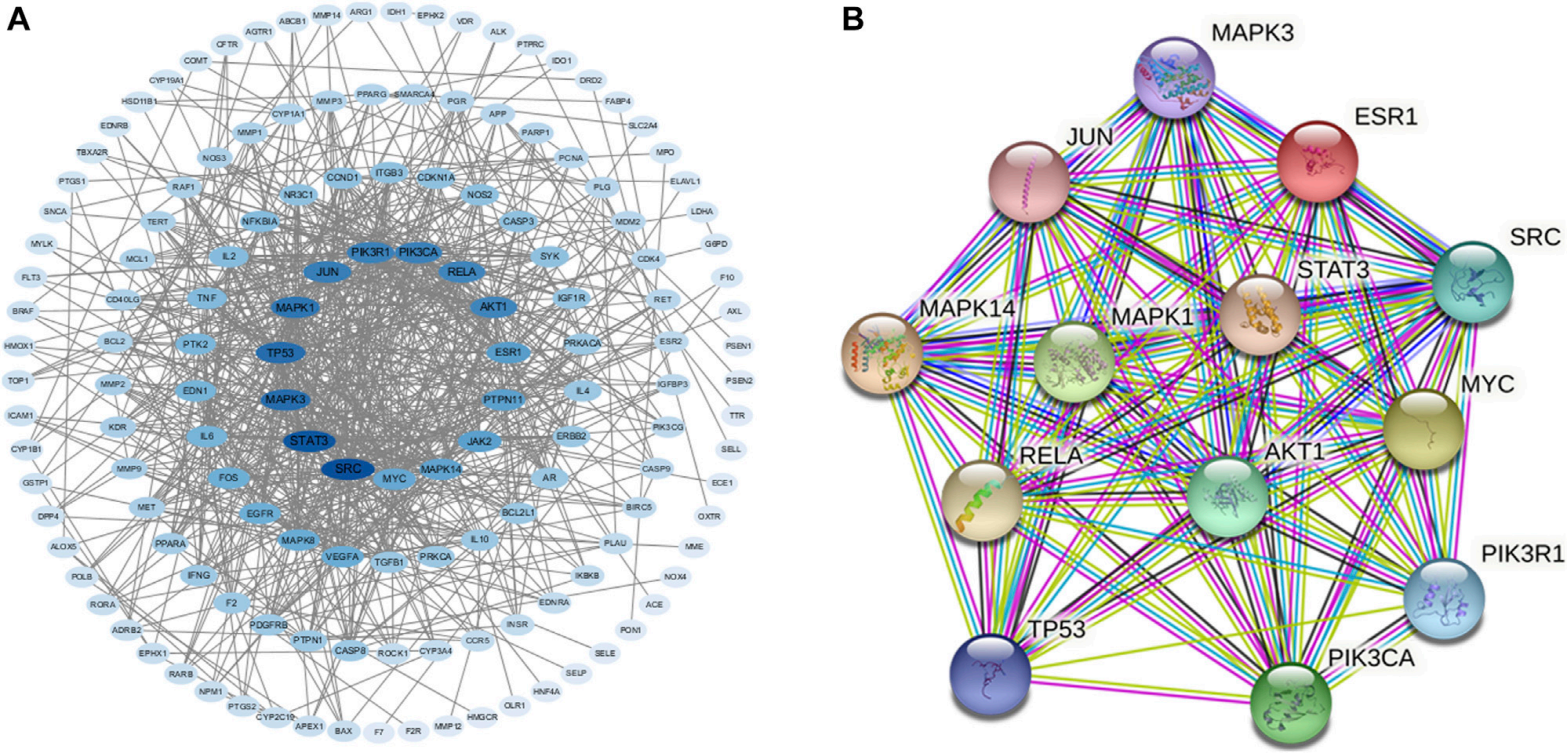
**(A)** PPI network of intersection protein targets. The size and color of the targets are based on the degree value. The larger and deeper the node is, the more important the target is. **(B)** PPI network of 13 core genes.

We used the STRING database to construct PPIs of these targets. As shown in Figure 2A, the PPI network has 147 nodes and 753 edges in total with the screening threshold as 0.9. In the network, the degree of a node represents the number of routes connected to the node. The larger the degree is, the stronger the interaction is. The size and color of the node are based on the degree. As shown in Figure 2A and Table 1, core genes in the network included SRC Proto-Oncogene, Non-Receptor Tyrosine Kinase (SRC), Signal transducer and activator of transcription 3 (STAT3), Mitogen-activated protein kinase 3 (MAPK3), Tumor protein P53 (TP53), Mitogen-activated protein kinase 1 (MAPK1), Jun Proto-Oncogene (JUN), phosphatidylinositol-4,5-bisphosphate 3-kinase, catalytic subunit alpha (PIK3KA), phosphoinositide-3-kinase regulatory subunit 1 (PIK3R1), RELA proto-oncogene, NF-kB subunit (RELA), AKT Serine/Threonine Kinase 1 (AKT1), Estrogen Receptor 1 (ESR1), Mitogen-activated protein kinase 14 (MAPK14), and MYC protooncogene (MYC). Figure 2B shows the PPI network of these 13 core genes.

**TABLE 1:** Designations and topological parameters of core genes in the PPI network.

	Gene symbol	Protein name	Degree	Betweenness centrality	Closeness centrality
1	SRC	SRC proto-oncogene, non-receptor tyrosine kinase	48	0.12100666	0.5087108
2	STAT3	Signal transducer and activator of transcription 3	46	0.09981434	0.48829431
3	MAPK3	Mitogen-activated protein kinase 3	40	0.05375253	0.49829352
4	TP53	Tumor protein P53	40	0.16505723	0.48504983
5	MAPK1	Mitogen-activated protein kinase 1	38	0.04985122	0.49491525
6	JUN	Jun proto-oncogene	36	0.07337968	0.50344828
7	PIK3CA	phosphatidylinositol-4,5-bisphosphate 3-kinase, catalytic subunit alpha	35	0.03772272	0.46794872
8	PIK3R1	phosphoinositide-3-kinase regulatory subunit 1	35	0.02263502	0.45625
9	RELA	RELA proto-oncogene, NF-kB subunit	34	0.06098774	0.50171821
10	AKT1	AKT Serine/threonine kinase 1	34	0.04208524	0.47402597
11	ESR1	Estrogen receptor 1	28	0.03979472	0.46794872
12	MAPK14	Mitogen-activated protein kinase 14	26	0.02354919	0.4591195
13	MYC	MYC protooncogene	25	0.03096713	0.45625

### Functional Annotations for Potential Therapeutic Targets of Dan-Shen-Yin

**FIGURE 3:**
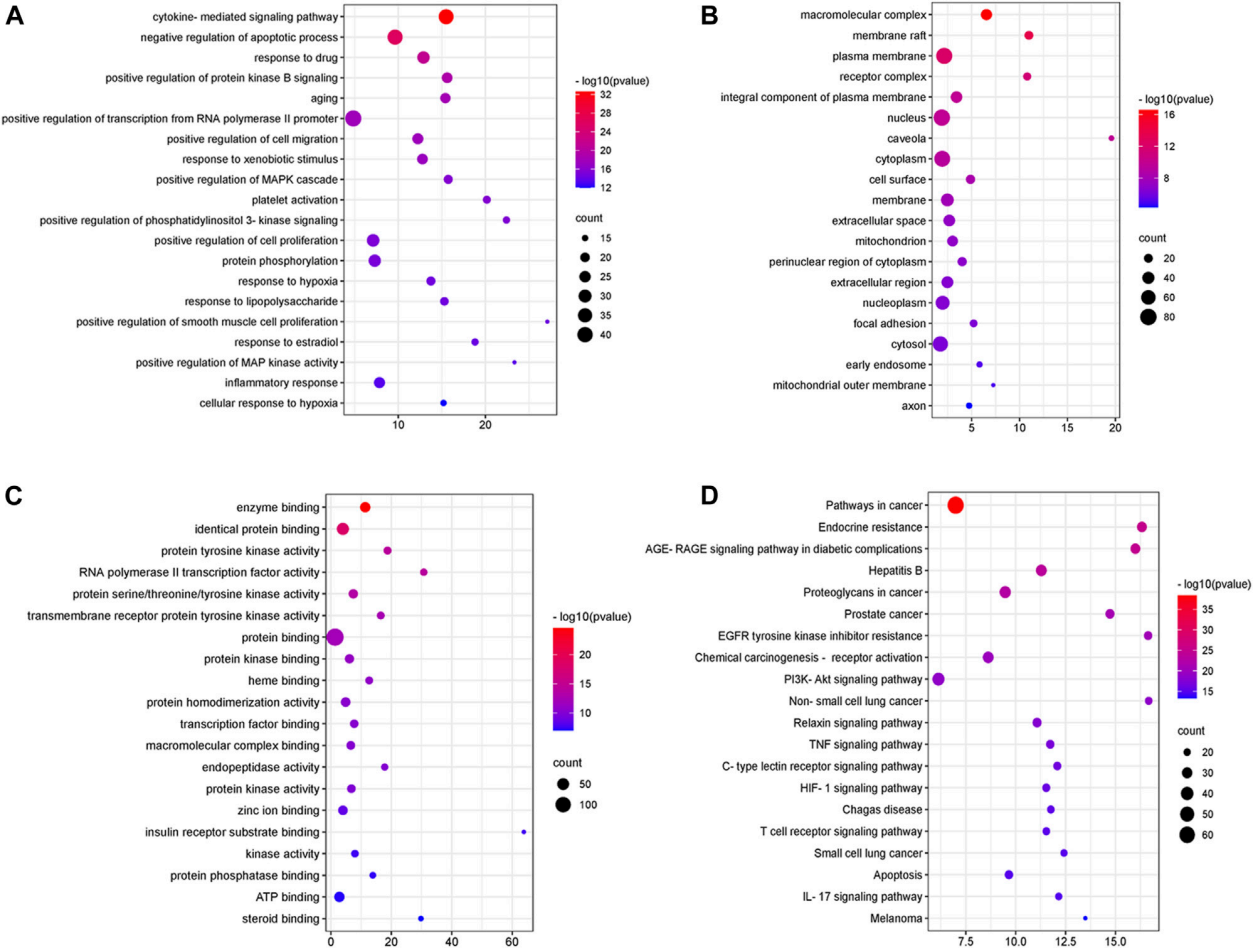
GO enrichment and KEGG pathway analysis of DSY targets. **(A)** “biological process” (BP) categories, **(B)** “cellular components” (CC) categories, **(C)** “molecular function” (MF) categories and **(D)** KEGG pathways.

For the 147 genes corresponding to potential therapeutic targets, DAVID was used to conduct functional annotation from different perspectives, respectively. There are 1,136 GO entries, of which 881 entries are related to biological processes, including negative regulation of apoptotic process, positive regulation of cell migration, positive regulation of cell proliferation, response to hypoxia and inflammatory response. 162 items are related to molecular function including enzyme binding, protein tyrosine kinase activity, protein binding, protein homodimerization activity, protein phosphatase binding and so on, and 93 cell components entries including macromolecular complex, membrane raft, plasma membrane, receptor complex, and so on (Figures 3A–C). According to the KEGG analysis, 179 pathways were screened (Figure 3D), including PI3K-Akt signaling pathway, TNF signaling pathway, HIF-1 signaling pathway, T cell receptor signaling pathway and so on.

### Construction of the Target-Function Network

**FIGURE 4:**
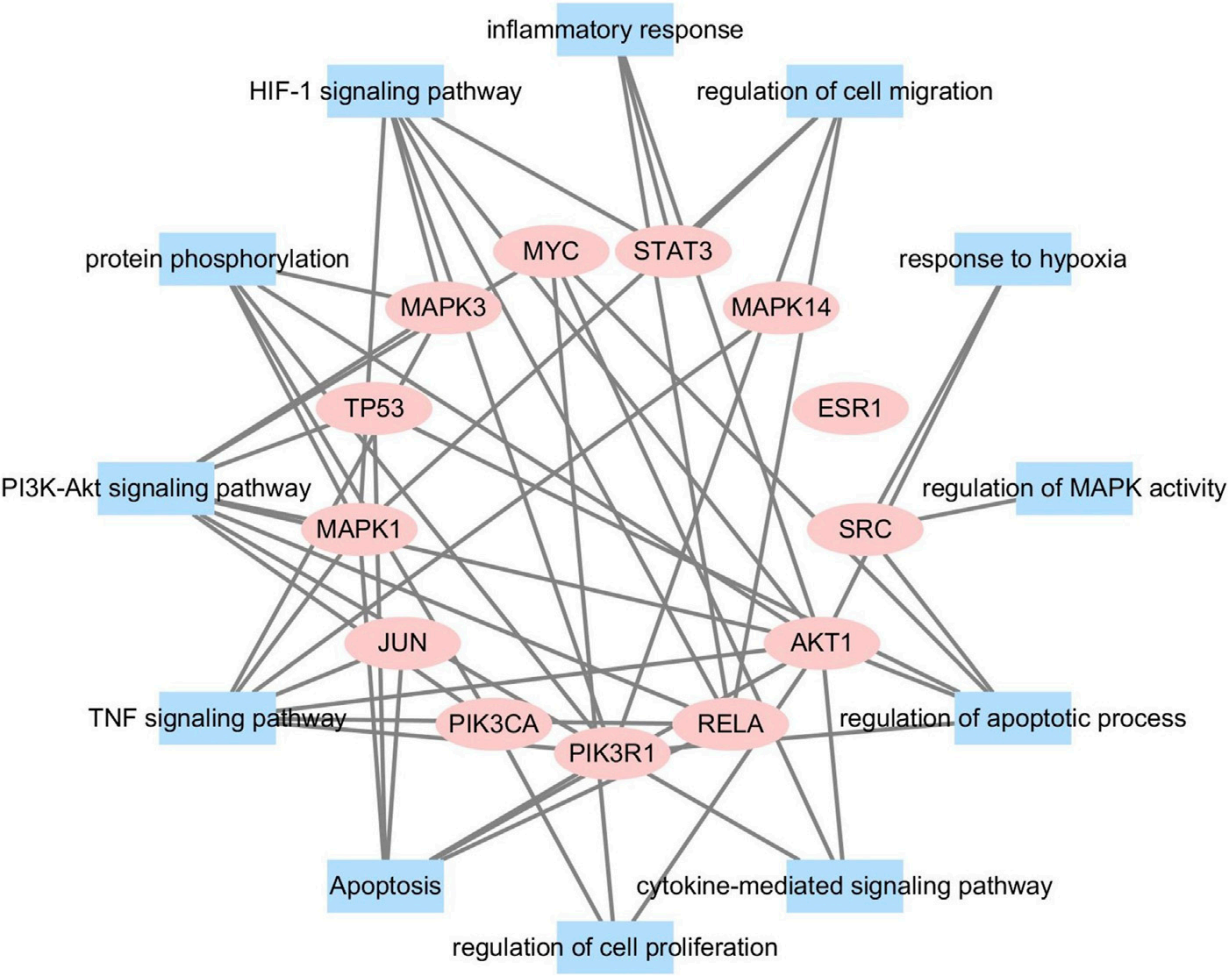
A functional module is linked to the target if the target is involved in that biological process or pathway.

We conducted further network analysis on several representative signal pathways and biological processes and constructed the target-function network. As shown in Figure 4, many targets were involved in multiple pathways simultaneously. For example, STAT3 belonged to “regulation of apoptotic process,” “positive regulation of cell migration,” and “HIF-1 signaling pathway.” AKT1 was involved in many biological processes and pathways such as “negative regulation of apoptotic process,” “proliferation of smooth muscle cell,” “response to hypoxia,” and “HIF-1 signaling pathway.” The above results indicate that DSY can exert an anti-HPH effect through multiple targets and pathways, so DSY might be suitable for the complex mechanism of pulmonary hypertension.

### Effect of Dan-Shen-Yin Treatment on Body Weight and Walk Distance of Hypoxia-Induced Pulmonary Hypertension Mice

**FIGURE 5:**
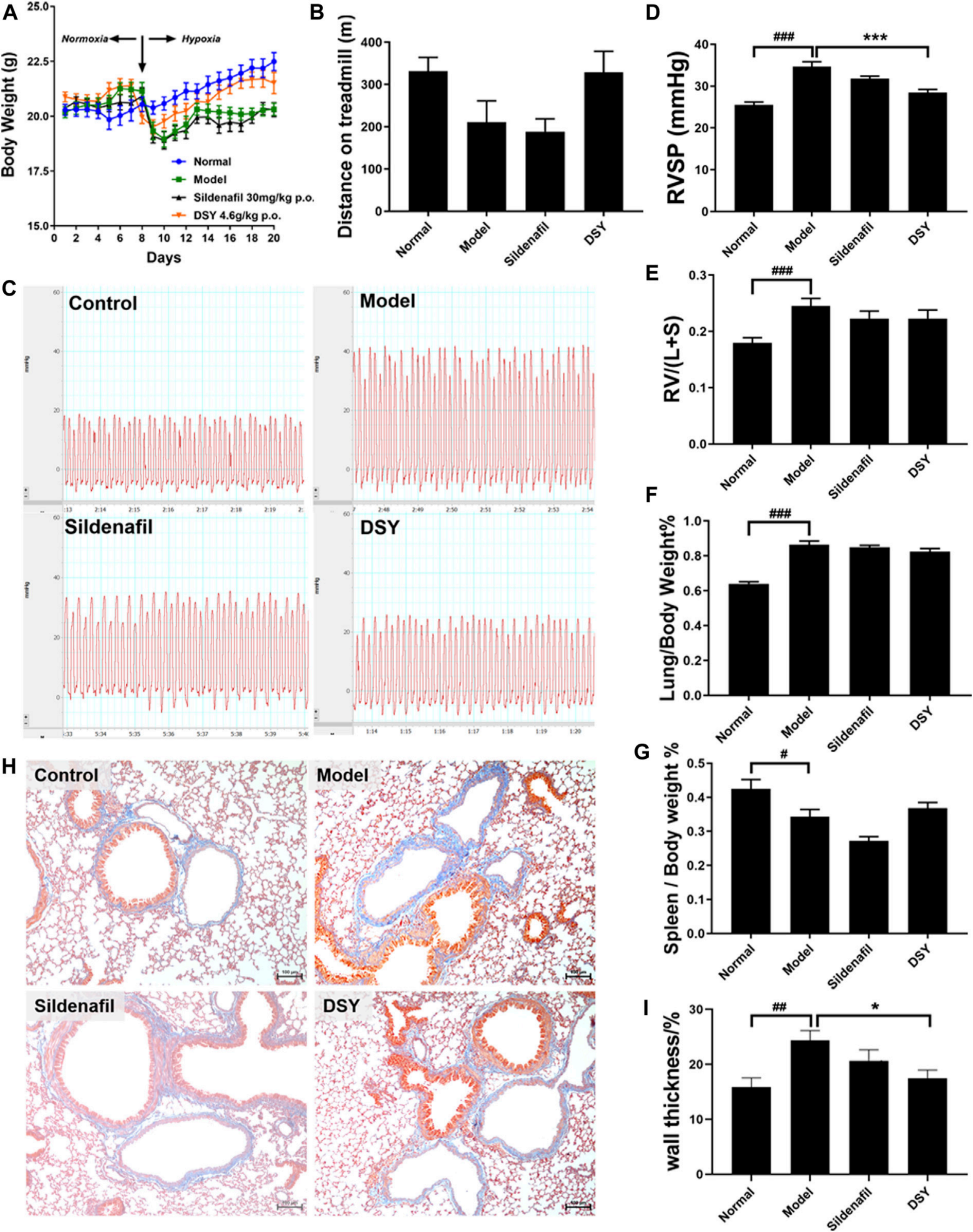
Effects of DSY treatment on HPH mice. **(A)** Body weight of mice in different groups. **(B)** Walking distance of mice until exhausted. **(C)** Representative images of RVSP waveform in different groups. **(D)** RVSP of mice in different groups. **(E)** Right ventricular hypertrophy index of mice in different groups. **(F)** Lung index of mice in different groups. **(G)** Spleen index of mice in different groups. **(H)** Representative images of masson staining of pulmonary arteries. The original magnification of the images was ×100. **(I)** Wall thickness of pulmonary arteries (%) in different groups. The data are expressed as the mean ± SEM (*n* = 5). #*p* < 0.05 vs. control group. ###*p* < 0.001 vs. control group. *p < 0.05 vs. model group. ***p < 0.001 vs. model group.

We monitored the body weight of mice everyday. The body weight of mice decreased after exposure to hypoxia, and then the growth slowed down. The positive drug sildenafil had no effect on body weight. DSY had an improving effect, but there was no significant difference (Figure 5A).

At the end of treatment, mice were placed on the treadmill and run until exhausted. The animal is considered exhausted if it stays on the battery lead plate for more than 10 s. Walk distance decreased in the model group (210.8 ± 50.24 vs. 331.6 ± 32.45 m). Sildenafil had no impact on walking distance, while treatment with DSY showed a tendency to extend the walking distance after exhaustion (Figure 5B).

### Dan-Shen-Yin Alleviated Hypoxia-Induced Increase in Right Ventricular Systolic Pressure

Typical RVSP waveform was shown in Figure 5C. A significant increase in RVSP was noted in hypoxia-treated mice compared with the normal mice (34.64 ± 1.16 vs. 25.53 ± 0.67 mmHg; *p* < 0.01). Treatment with sildenafil exhibited a decrease in RVSP, but there was no significant difference. Treatment with DSY demonstrated a prominent reduction of RVSP to 28.44 ± 0.77 mmHg from 34.64 ± 1.16 mmHg in the model group (Figure 5D).

### Effect of Dan-Shen-Yin Treatment on Organ Indices of Hypoxia-Induced Pulmonary Hypertension Mice

The weight ratio of RV/LV + S is typically calculated as an indicator of RVH. Hypoxia caused a significant increase in the RV/LV + S ratio (0.25 ± 0.01 vs. 0.18 ± 0.01; *p* < 0.001, Figure 5E). Treatment with DSY exhibited a decrease in RV/LV + S ratio to 0.22 ± 0.03 compared with the model group (0.25 ± 0.01). It suggested that DSY had a certain tendency to inhibit RVH, but there was no significant difference.

Similarly, the lung/body weight ratio was significantly increased in the model group (0.86 ± 0.02) compared with the control group (0.64 ± 0.01; *p* < 0.01, Figure 5F). The DSY group exhibited a decreasing tendency in this ratio to 0.82 ± 0.02 compared with the model group, but with no significant difference.

Moreover, the spleen/body weight ratio was significantly decreased in the model group (0.34 ± 0.02) compared with the control group (0.42 ± 0.01; *p* < 0.05, Figure 5G). Treatment with sildenafil decreased the ratio to 0.27 ± 0.01. The DSY group exhibited an increase to 0.37 ± 0.02 compared with the model group, but with no significant difference.

### Effect of Dan-Shen-Yin Treatment on Vascular Remodeling Induced by Hypoxia

Remodeling of pulmonary arteries was determined by Masson trichrome stain (Figure 5H). The wall thickness of pulmonary arteries (%) in the model group was significantly increased (0.24 ± 0.02 vs. 0.15 ± 0.02; *p* < 0.01), which was reversed by DSY administration from 0.24 ± 0.02 to 0.17 ± 0.01 (*p* < 0.05, Figure 5I).

### Effect of DSY on the Cell Viability Under a Hypoxic Environment

**FIGURE 6:**
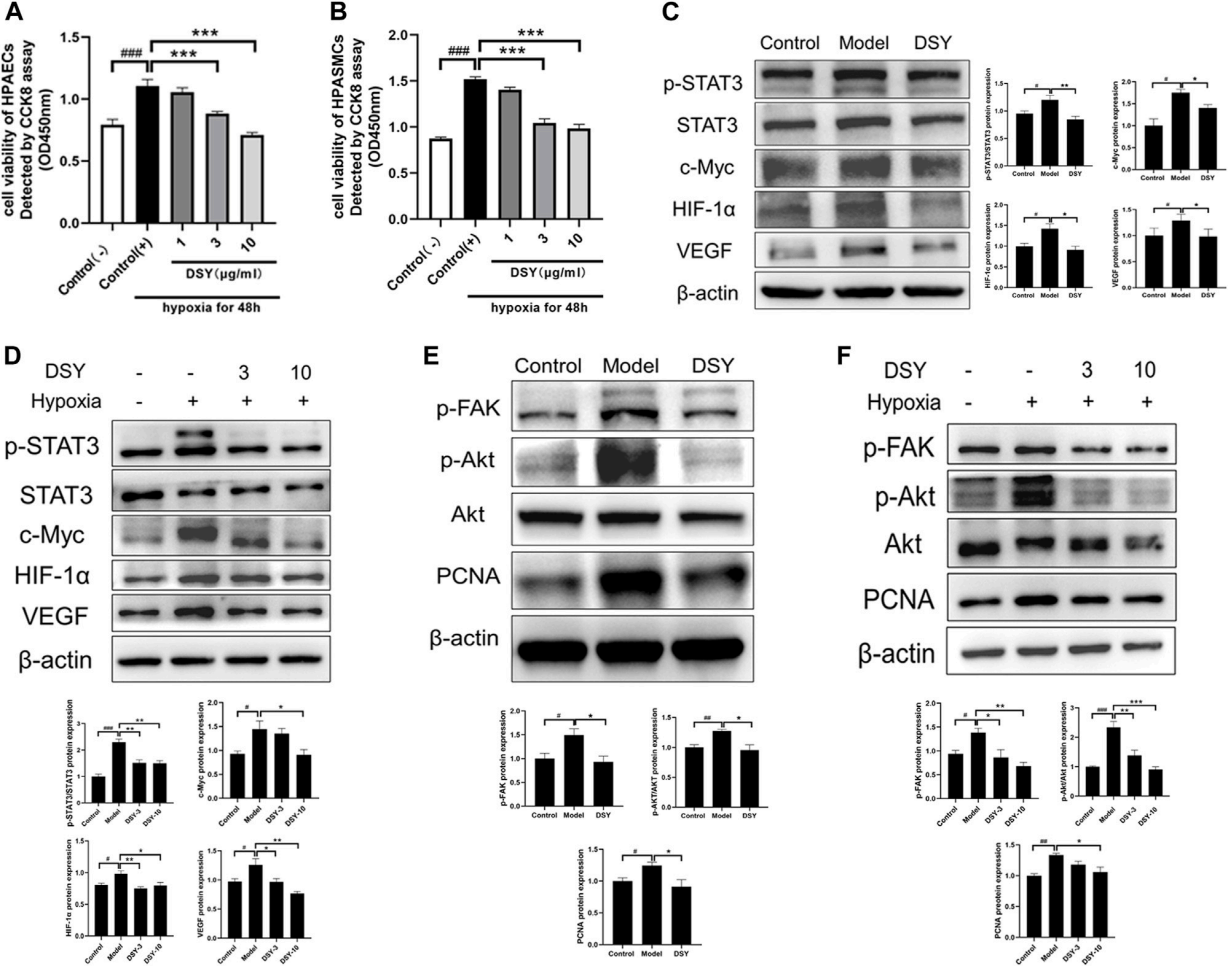
**(A)** Cell viability of HPAECs after incubating in hypoxia environment for 48 h detected by CCK8 assay. **(B)** Cell viability of HPASMCs after incubating in hypoxia environment for 48 h detected by CCK8 assay. **(C)** Effect of DSY on STAT3-HIF-VEGF *in vivo*. **(D)** Effect of DSY on STAT3-HIF-VEGF in HAPECs. **(E)** Effect of DSY on FAK-AKT *in vivo*. **(F)** Effect of DSY on FAK-AKT in HPASMCs. The data are expressed as the mean ± SEM (*n* = 3–5). #*p* < 0.05 vs. control group. ##*p* < 0.01 vs. control group. ###*p* < 0.001 vs. control group. *p < 0.05 vs. model group. **p < 0.01 vs. model group. ***p < 0.001 vs. model group.

To investigate the effect of DSY on pulmonary artery cells, the cells were pretreated with DSY (1, 3 and 10 μg/ml) for 2 h before incubating in hypoxia (1% O_2_) for 48 h. At the end of the experiment, the cell viability was detected by CCK8 assay. As shown in Figures 6A,B, the value of OD450 nm was significantly higher in the hypoxia groups than in the normal group, indicating that hypoxia could cause the proliferation of HPAECs and HPASMCs. The treatment of the DSY (3 and 10 μg/ml) significantly inhibited the proliferation, while the other group did not show any obvious effect.

### Dan-Shen-Yin Attenuated Signal Transducer and Activator of Transcription 3 Phosphorylation and HIF-1α Expression in Hypoxia-Treated Mice and HPAECs

To explore the possible mechanisms underlying the protective effects of DSY on hypoxia-induced vascular remodeling, we examined the phosphorylation of STAT3 (p-STAT3) protein levels and HIF-1α protein levels in lung tissues and HPAECs. As shown in Figure 6C, hypoxia upregulated the expressions of phosphorylation of STAT3 (p-STAT3), c-Myc, HIF-1α, and VEGF, which were diminished by DSY. Similar results were observed in HPAECs (Figure 6D). These results implied that DSY is responsible for the expression of HIF-1α, VEGF, p-STAT3, and the expression of the STAT3 target gene c-Myc. Moreover, a decrease in the expression of HIF-1α and p-STAT3 by DSY treatment might play a vital role in preventing vascular remodeling in hypoxia-treated mice.

### Dan-Shen-Yin Inhibited FAK/AKT Signaling Pathway in Hypoxia-Induced Pulmonary Hypertension Mice and Hypoxia-Treated HPASMCs

AKT was shown to be an important target in network pharmacology analysis. The FAK/AKT pathway has previously been reported to promote the proliferation of HPASMCs (Jia et al., 2017). To determine whether this pathway was activated in HPH, we examined FAK phosphorylation, AKT phosphorylation, and PCNA in lung tissues and HPASMCs. Exposure to hypoxia increased the phosphorylation of both FAK and AKT, which were decreased by DSY treatment. PCNA also increased in the model group and diminished by DSY (Figures 6E,F). These observations support that DSY might inhibit PASMC proliferation through FAK/AKT pathway.

## Discussion

The past 2 decades have witnessed the coming of network science as the central paradigm behind some of the most fascinating discoveries of the 21st century (Gosak et al., 2018). Increasing massive data has led to the emergence and rapid development of network pharmacology, which is an interdisciplinary field that combines traditional pharmacology, structural biology, computational science, and a series of OMICs approaches (Chen et al., 2019). For complex diseases, network pharmacology has shown unique advantages. Thus, we employed this method to explore the anti-HPH effect of DSY.

Firstly, the targets of DSY and genes related to HPH were obtained from multiple databases.78 compounds of DSY were screened out. The 190 intersection genes of DSY and HPH may be the potential therapeutic targets of DSY against HPH. GO and KEGG pathway analysis were conducted using DAVID, and networks were constructed using Cytoscape 3.7.2. When exploring the mechanism of action of DSY, we focused on analyzing the biological processes and signaling pathways associated with cardiovascular diseases, especially HPH. From the KEGG and GO biological process enrichment, the highly enriched pathways of DSY were associated with oxidative stress (“HIF-1 signaling pathway,” and “response to hypoxia”), cell proliferation (“negative regulation of apoptotic process,” and “positive regulation of cell proliferation”), inflammation (“response to lipopolysaccharide,” “inflammatory response”), and PI3K-Akt signaling pathway.

The pathological mechanism of HPH is very complicated. Consistent with network analysis, previous studies have demonstrated that the development of pulmonary hypertension is highly related to oxidative stress (Mikhael et al., 2019; Li et al., 2020; Türck et al., 2020). Our preliminary experiments show that DSY has no effect on PH induced by monocrotaline (results are not shown), but is effective for HPH. Considering the differences between the two types of PH, we speculate that the anti-HPH effect of DSY may be related to HIF-1α. Hypoxia-inducible factor (HIF) is an oxygen-dependent transcriptional activator, which plays a pivotal role in angiogenesis. HIF-1 activation is a mediator of physiological and pathophysiological responses to hypoxic conditions (Semenza, 2000), which is regulated by various post-translational modifications, hydroxylation, acetylation and phosphorylation (Choudhry and Harris, 2018). It is confirmed that HIF-1α activation is involved in vascular remodeling in pulmonary hypertension (Luo et al., 2019; Pullamsetti et al., 2020). The signal transducer and activators of transcription (STAT) protein family regulate diverse cellular processes including growth and survival, and is frequently deregulated in cancer (Zou et al., 2020) and several other disorders. The role of STAT3 in PH has been suggested in 2007 (Masri et al., 2007) and strengthened in recent years (Paulin et al., 2012; Cotroneo et al., 2015). STAT3 can induce the expression of factors that contribute to cellular proliferation and survival, such as Pim1 and Survivin (Paulin et al., 2012); immunosuppression, such as TGFβ and IL-10, and angiogenesis, such as HIF-1α and VEGF (Behera et al., 2010; Johnson et al., 2018; Zhang et al., 2019). HIF-1α is a prominent transcription target for STAT3 (Papadakis et al., 2010). Besides, STAT3 was proposed to be required for the function of the HIF-1α complex (Qian et al., 2019). Numerous investigators have reported that the STAT3/HIF-1α pathway is closely associated with tumorigenesis and the progress of various tumors (Tong et al., 2020; Wang et al., 2020; Zhao et al., 2020), but there is no report on PH before.

**FIGURE 7:**
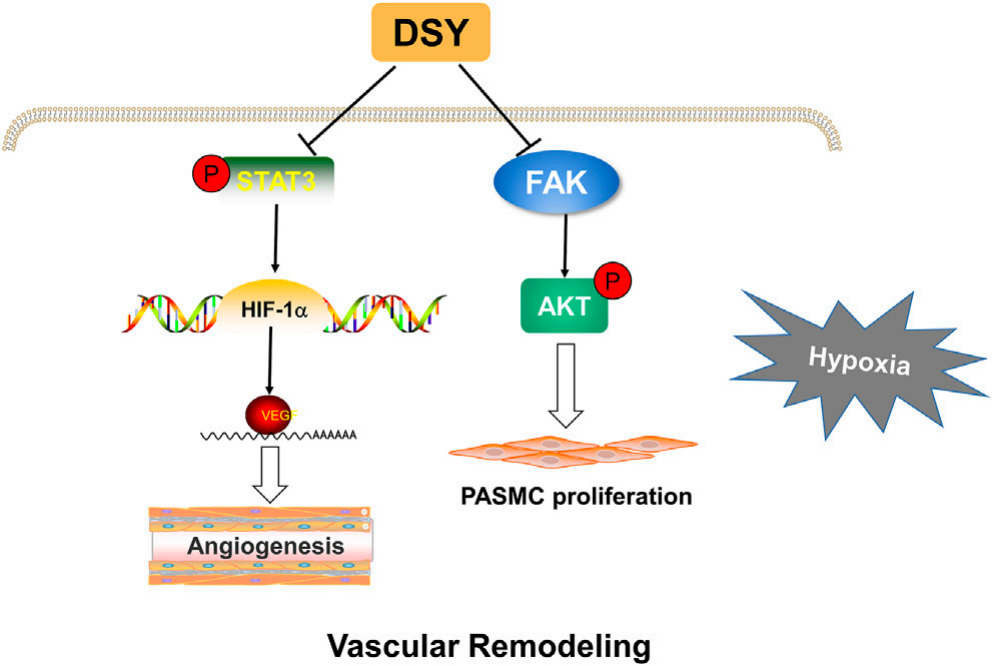
Proposed mechanisms of DSY against hypoxia-induced pulmonary hypertension. DSY inhibits STAT3/HIF-1α/VEGF pathway, thereby inhibiting pulmonary angiogenesis. DSY suppresses PASMC proliferation through FAK/AKT pathway.

Focal adhesion kinase (FAK) is a cytoplasmic tyrosine kinase that acts as a mediator of cell signaling downstream of growth factor and cytokine receptors (Paul et al., 2020). FAK has been reported to control tumor cell survival, proliferation, and migration (Paul et al., 2020; Wu et al., 2020). Kinase function of FAK has been shown to activate the PI3K-Akt pathway, which can protect cells from apoptosis and promote survival (Sulzmaier et al., 2014). Abnormal proliferation of pulmonary artery smooth muscle cells is one of the pathological mechanisms leading to vascular remodeling (Thenappan et al., 2018). It is reported that FAK/AKT suppression can attenuate the proliferation of pulmonary artery smooth muscle cells (Jia et al., 2017). Considering that AKT was highly expressed in network pharmacology analysis, we investigated whether FAK/AKT signaling pathway was involved in DSY against HPH. Increased phosphorylation of FAK and AKT and the expression of PCNA were suppressed by DSY treatment, indicating that DSY may inhibit smooth muscle cell proliferation through the FAK/AKT pathway. As a multitargeted drug composed of complicated components, DSY may exert its anti-pulmonary hypertension effect through various pathways (Figure 7).

The current study, which investigated the impact and mechanism of DSY on HPH, achieved some findings. Firstly, DSY treatment significantly attenuated the increases in the RVSP and pulmonary artery remodeling induced by hypoxia. Next, DSY therapy downregulated HIF-1α expression and STAT3 phosphorylation in pulmonary tissues, thus implying its possible role in the HPH model, which is consistent with the network pharmacology analysis.

Our study has some limitations. Firstly, due to the large number of databases and the constantly updated data, the signaling networks we construct may not cover all known protein-protein interactions. Next, only one dose of DSY was investigated in the *in vivo* experiment. As a well-known traditional Chinese formula, DSY is widely used in clinical practice for the treatment of coronary heart disease. Several studies have reported that it can effectively treat myocardial ischemia/reperfusion injury, ischemic myocardial injury, and angina pectoris. However, there is no related reports on DSY in the treatment of PH. Considering that excessive dose studies are meaningless, we chose the clinically equivalent dose to explore whether DSY can alleviate HPH. The clinically equivalent dose can ensure the safety of the drug and avoid the impact of adverse reactions. Fortunately, we found that it has a preventive effect on HPH. Whether DSY is dose-dependent in the treatment of HPH remains to be studied. Notably, it is unclear whether the findings in the HPH mouse model can be generalized to humans. Therefore, comprehensive *in vivo* and *in vitro* studies are required to test this hypothesis in the future.

Taken together, network pharmacology provides a new approach for exploring the targets and mechanisms of TCMs, which are composed of multiple and complex ingredients. The deductions drawn from the computational analysis are, in part, validated by our subsequent *in vivo* experiments. The results demonstrate that DSY can prevent the development of HPH in a hypoxia-induced mouse model and alleviate pulmonary vascular remodeling. Moreover, inhibition of the STAT3-HIF-1α signaling pathway and FAK-AKT pathway in the lung might serve as mechanisms involved in the therapeutic effects of DSY. With complex ingredients, DSY has shown a multitargeted effects on pulmonary hypertension. Multi-target therapy may be an important strategy for the treatment of pulmonary hypertension in the future.

## Data Availability

The original contributions presented in the study are included in the article/Supplementary Material, further inquiries can be directed to the corresponding authors.
